# Supplementary material for: Learning curve and surgical outcome of robotic assisted colorectal surgery with ERAS program
Source: Sci Rep. 2022 Nov 29;12:20566. doi: 10.1038/s41598-022-24665-w (PMC9709162; doi:10.1038/s41598-022-24665-w)
Supplement: Supplementary file 2 — Supplementary Information 2. [file 41598_2022_24665_MOESM2_ESM.zip › RAL Raw data-Ñ[▒K/100/HS_ñw╝╨░O▒Kñσ.pdf]

正式摘要(電子簽章：000000221419820)	□【讀取】憑證	
W83 / W83 □英文標題(注意:無英文姓名-請補病人基本檔!)		

臺中榮民總醫院
出院病歷摘要

轉入號： 轉出號：

[redacted]

(8)流水編號	
(9)入院日期	110/07/22 (2021) 直腸外科 W83 - 093
(10)轉科日期	
(11)出院日期	110/07/26 (2021) 住院天數計 4 日

診斷

- 入院：Rectal adenocarcinoma, moderately-differentiated, s/p Sigmoid colonoscopic biopsy on 2021/07/20
- 出院：R-S colon adenocarcinoma, with right ovary involved and uterine seeding, s/p Sigmoid colonoscopic biopsy on 2021/07/20, s/p Robotic LAR + Right oophorectomy on 2021/07/22

主訴

- diarrhea for two months, with bloody stool

病史

- This 68-year-old woman, who received C/S for once for pregnancy, denied other systematic disease.

  She presented with diarrhea for two months, with bloody stool. She visited Cheng-Chin hospital at first, where IBS was diagnosed. She visited our CRS OPD on 2021/07/20. PE showed a palpable tumor from rectum. Tumor markers was elevated, with CEA 67.01. Sigmoid colonscopy on 2021/07/20, up to rectum, found a rectum tumor, s/p biopsy. Pathology showed adenocarcinoma, moderately-differentiated. Abdominal CT on 2021/07/21 reported rectal ca, without significant enlarged lymph node in retroperitoneum or bilateral inguinal regions.

  For rectal adenocarcinoma, she was admitted for Robotic LAR scheduled on 2021/07/22.

體檢發現

- PLAPBLE TUMOR
  ELEVATED CEA 67.01
  - General appearance :
    - easy looking , conscious clear , without cardiopulmonary distress
    - Vital signs自動帶入date 體溫(BT) 脈搏(P) 呼吸(R) 血壓(BP) 身高(BH) 體重(BW) BMI
      20210722 35.7℃ 91次/分 17次/分 127/81mmHg 152cm 49.7kg 21.5
    - Vital signs: B.P. 127/81 mmHg , P.R. 91 /min , R.R. 17 /min , B.T. 35.7 ℃
  - HEENT :
    - Eyes: no anemic conjunctiva, no icteric sclera, normal EOM
    - ENT: no otorrhea, no rhinorrhea, no erythematous mucosa, no injected tonsil, no deformity
  - Neck: supple, freely movable, trachea midline,no deformity, no engorged jugular vein , no palpable thyroid or lymph node enlargement
  - Chest:
    - Inspection: Grossly normal, symmetric and fully expansion, no accessory muscle used, no operation scar, no spider angiomata, no gynecomastia
    - Palpation: no palpable mass or nodule, no subcutaneous emphysema, no tenderness
    - Ausculation: clear breathing sound
  - Heart:
    - Palpation: no thrill, no heave
    - Percussion: normal heart size
    - Ausculation: regular heart beat, normal S1, S2, no gallop, no murmur
  - Extremities: No deformity, full ranged of motion without limitation, no clubbing finger or

- nail cyanosis, no pitting edema, no ulcer or pigmentation, no obvious skin lesion, no varicose vein, no bone, joint or muscle tenderness

CRS CONDITION

- Karnofsky performance status: (100) Normal, no complaints, no evidence of disease
- PE of Abdomen:
- CEA: 67 ng/ml
- Imaging Studies:
- Colonoscope report: Sigmoid colonscopy on 2021/07/20, up to rectum, found a rectum tumor, s/p biopsy. Pathology showed adenocarcinoma, moderately-differentiated.
- Sono , CT of abdomen and chest Films:Abdominal CT on 2021/07/21 reported rectal ca, without significant enlarged lymph node in retroperitoneum or bilateral inguinal regions.
- Figures:
  1. colon & rectum

手術日期及方法

- 手術日期及方法20210722 Robot Assisted Colectomy(L)

住院治療經過

- After operation with Robotic LAR on 2021/07/22, the patient was under post-operative care and pain control under PCA use. Self-urination was smoothly after removal of Foley's catheter on 7/24. The patient tolerated clear liquid diet and then Bland No.II diet since 7/24, without N/V, abdominal pain or discomfort. The wound was clean and dry, without infectious signs. Under relative stable condition, the patient was discharge with OPD follow up.

合併症

一般檢查

- 累積報告：

DATE	TIME	ALB	TP	BIL.T	BIL.D	ALKP	AST	ALT	LDH	AMY	LIPASE	NH3	Ferritin	UA
1100722	1921	3.3		0.54	0.14	75	16	8						
1100726	0856			0.56			42	27						

DATE	TIME	WBC	RBC	HGB	HCT	MCV	PLT	BLST	NEUT	BAND	SEG	LYM	MON	EOS	BAS
1100722	1920	11620	3.74	9.8	31.2	83.4	247		93.1			5.2	1.6	0.0	0.1
1100726	0918	10450		10.0	32.6	86.5	273		85.0			9.2	5.4	0.3	0.1

DATE	TIME	NA	K	CL	BUN	CREAT	eGFR	CA	Mg	P	CRP	ESR	PCT	D-Dimer	LACTATE
1100722	1921	139	3.0	104	8	0.60	105.66	8.2							
1100726	0856	143	3.5		7	0.60	105.66	8.2							

DATE	TIME	GLUCOSE	HbA1C	CHOL	TG	HDL	LDL	KETONE	CK	CKMB	TRO-T	PT-P	APTT-P	NT-pro BNP
1100722	1920	136												

特殊檢查

病理報告

其它

出院時情況

- 3.改門診治療

出院指示

藥名	劑量	劑量單位	用法	頻次	數量	數量單位	現況	天數	單位
Mag. Oxide tab	2	TAB	PO	BIDPRN	28	TAB	TH	7	天
(口)Takepron OD tab 30mg	1	TAB	PO	QDAC	7	TAB	TH	7	天
Ultracet tab	1	TAB	PO	Q6H	28	TAB	TH	7	天

出院計劃

主治醫師 2062F 蔣鋒帆 / 住院醫師 2345B 陳映晴

正式摘要(電子簽章：**000000221419820)**

		W83 / W83				

doctorRank:B
